# Is the Risk of HIV Acquisition Increased during and Immediately after Pregnancy? A Secondary Analysis of Pooled HIV Community-Based Studies from the ALPHA Network

**DOI:** 10.1371/journal.pone.0082219

**Published:** 2013-12-26

**Authors:** Milly Marston, Marie Louise Newell, Amelia Crampin, Tom Lutalo, Richard Musoke, Simon Gregson, Constance Nyamukapa, Jessica Nakiyingi-Miiro, Mark Urassa, Raphael Isingo, Basia Zaba

**Affiliations:** 1 London School of Hygiene and Tropical Medicine, London, United Kingdom; 2 Africa Centre for Health and Population Studies, University of KwaZulu-Natal, Somkhele, South Africa; 3 Faculty of Medicine, Faculty of Social and Human Sciences, University of Southampton, Southampton, United Kingdom; 4 Karonga Prevention Study, Chilumba, Malawi; 5 Rakai Health Sciences program, Kalisizo, Rakai, Uganda; 6 Imperial College London, London, United Kingdom; 7 Biomedical Research and Training Institute, Harare, Zimbabwe; 8 Medical Research Council, Entebbe, Uganda; 9 TAZAMA project, National Institute of Medical Research, Mwanza, Tanzania; University of Athens, Medical School, Greece

## Abstract

**Background:**

Previous studies of HIV acquisition in pregnancy have been in specific population groups, such as sero-discordant couples which have shown an increased risk of HIV acquisition during pregnancy and studies of sexually active women where the results have been ambiguous. However these studies are unable to tell us what the overall impact of pregnancy is on HIV acquisition in the general population.

**Methods:**

Data from six community-based HIV cohorts were pooled to give 2,628 sero-conversions and a total of 178,000 person years of observation. Multiple imputation was used to allow for the uncertainty of exact sero-conversion date in surveillance intervals greater than the length of a pregnancy. Results were combined using Rubin’s rules to give appropriate error bounds. The analysis was stratified into two periods: pre- and post- widespread availability of prevention of mother-to-child HIV transmission services. This allows us to assess whether there is reporting bias relating to a person’s knowledge of their own HIV status which would become more widespread in the latter time period.

**Results:**

Results suggest that women while pregnant have a lower risk of acquiring HIV infection over all periods (HRR 0.79, 95%CI 0.70-0.89) than women who were not pregnant. There is no evidence for a difference in the rate of HIV acquisition between postpartum and non-pregnant women (HRR 0.92 95%CI 0.84-1.03).

**Discussion:**

Although there may be immunological reasons for increased risk of HIV acquisition during pregnancy, at a population level this study indicates a lower risk of HIV acquisition for pregnant women. Pregnant women may be more likely to be concordant with their current sexual partner than non-pregnant women, i.e. either already HIV positive prior to the pregnancy or if negative at the time of becoming pregnant more likely to have a negative partner.

## Introduction

Fertility rates are high in many sub-Saharan African countries and, thus, a significant proportion of woman-years are spent pregnant [1]. Evidence regarding the risk of acquisition of HIV infection at and shortly after the time of pregnancy is conflicting [2-8]. An increased risk of HIV acquisition in pregnant women has implications for health services as the increased viral load in acute infection would expose the fetus to higher risk of in utero mother-to-child transmission [9]. This would also have implications for HIV epidemic modelling as estimates for paediatric HIV would need to be revised upwards.

A number of prospective studies from Eastern and Southern Africa have assessed the risk of HIV incidence during pregnancy. A multisite study of sero-discordant couples found that HIV incidence was, in univariate analysis, two-fold higher in pregnant than in not-pregnant women; however, after adjusting for age, any unprotected sex in last month, and contraceptive use, the risk difference was reduced and no longer statistically significant [5]. A similar study in Uganda restricted to married sero-discordant couples reported a non-significant increase in the HIV acquisition rate in pregnant women [2]. Other studies included women regardless of the partners’ HIV status; in a Ugandan study of sexually-active women the risk of HIV-1 acquisition was doubled during pregnancy [2]. However, in an HIV prevention trial enrolling women from a number of health services and community venues in southern Africa there was no increased risk of HIV-1 in pregnant women [7]. A study in Uganda and Zimbabwe, in which women from family planning sites were enrolled, found no increased risk of HIV acquisition in pregnant women in the pooled analysis overall, and actually showed some evidence of a protective pregnancy effect in one of the sites in the study after adjusting for covariates [4]. Further studies have shown a possible increased HIV incidence during pregnancy [10-12], others showed a risk comparable to the general population of a similar age [13,14]. 

A number of studies have investigated HIV incidence in the postpartum period, again with somewhat conflicting results. In Malawi, a prospective study of women enrolled after delivery found HIV acquisition was increased in the first year postpartum decreasing subsequently [8]; this was also the case in Zimbabwe [6] and Rwanda [3]. The authors of the latter study suggested that the decrease could be partly due to a cohort selection bias with those remaining uninfected for longer having a lower risk of infection . Other studies have not reported an increased risk in the postpartum period [2,15]. 

The rate of HIV acquisition and differences between pregnant, postpartum and non-pregnant women at a population level will depend not only on the risk of infection per sexual act with an HIV positive partner, but also on the level of discordance in pregnant and non-pregnant couples and the differences in sexual behaviour between these groups. Therefore results from the studies outlined above cannot be generalised to the general population.

Population-based HIV cohort studies are ideally placed to provide generalisable estimates of the risk of HIV during pregnancy in the community; this paper uses data from six such cohorts from eastern and southern Africa. We aim to assess the population-level HIV incidence during pregnancy and the post-partum period, adjusting for age. The results will inform organisations that provide estimates to health services providers. 

## Methods

### Data

Data come from six sites: Karonga (Karonga prevention study), Kisesa (TAZAMA), Masaka (UK Medical Research Council and Uganda Virus Research Institute), Rakai (Rakai Health Sciences), Manicaland (Imperial College London and the Biomedical Research and Training Institute) and uMkhanyukude (Africa Centre). Data collection was sufficiently similar to allow pooled analyses, with allowance for unobserved heterogeneity between sites.

The Karonga Demographic Surveillance Study (DSS) is located in rural northern Malawi; it was established in 2002 and has a total population of around 35,000, population-based HIV testing in the DSS was undertaken in four annual rounds from 2007-2011 [16] and average adult HIV prevalence between these dates was 8% [17]. The Kisesa cohort study is situated in rural north-west Tanzania, it was established in 1994 and has a population of around 30,000 it contains a small trading centre located on the main road from Mwanza town to the border of Kenya which runs through the centre of the study area, average HIV prevalence between 1994 and 2010 was 6% [18,19]. The Masaka DSS is located in rural south west Uganda and was established in 1989. Its initial population was around 10,000 which then increased to 18,000 when 10 villages were added to the census area [20]. Average HIV prevalence between 1989 and 2011 was 8% [21]. The Rakai Health Sciences Program runs the Rakai Community Cohort Study (RCCS), with an adult population of between 12,000-16,000. For this analysis, data were collected from 1999 with 2002/3 adult HIV prevalence reported to be 11.4 % [22]. The Manicaland study was established in 1993. A prospective household census (population size approximately 37,000) and general population cohort survey (10,000-12,000) were initiated in 12 locations spread across three districts in 1998, with follow-ups being conducted every 2 or 3 years. They comprise two small towns, four agricultural estates, two roadside settlements and four subsistence farming areas. Overall adult HIV prevalence has fallen in these areas from 24% in the late 1990s to 14% at the end of the 2010s [23]. The Africa Centre Surveillance study was established in 2000 in uMkhanyakude, in rural KwaZulu-Natal, South Africa; each round covers approximately 90,000 resident and non-resident household members in approximately 12,000 households, with a key-household respondent [24], an individual HIV surveillance for resident adults (≥15 years) was added in 2003 and adult HIV prevalence in 2012 was around 28% and annual incidence in the 15-50 year age group for women was about 5% [25]. 

### Ethics statement

Each of the six sites contributing data to the pooled analysis has received ethical clearance from the appropriate local ethics review bodies, and from the corresponding Institutional Review Boards for studies which had collaborating partnerships with Northern Universities. 

#### uMkhanyakude

Annually re-certified ethics permission for the Africa Centre DSS and nested individual HIV surveillance among consenting adults obtained from the Biomedical Research Ethics Committee at the Nelson Mandela School of Medicine, University of KwaZulu-Natal. Detailed written informed consent obtained for participation in the HIV surveillance. 

#### Karonga

Ethical approval granted by the National Health Sciences Research Committee of Malawi and the ethics Committee of the London School of Hygiene and Tropical Medicine. Written informed consent obtained for HIV testing. 

#### Kisesa

Ethical approval for each survey round of the Kisesa cohort study granted by the Tanzanian Medical Research Coordinating Committee and the Ethics Committee of the London School of Hygiene and Tropical Medicine. Prior to 2006, verbal consent obtained directly from all study participants (aged 15 and over), due to low literacy rates among the study population. Consent witnessed and documented for each study participant by a member of the sero-survey team. From 2006 onward, consent was again obtained directly from all study participants, however written consent option introduced, for those able to provide this. 

#### Manicaland

All respondents (all aged 15 years and older) provided written informed consent at each survey round prior to completing survey and providing a blood sample for HIV testing. For respondents under age 18 years, written informed consent was also provided by parent/guardian. Ethical approval for Manicaland HIV/STD Prevention Project provided by Medical Research Council of Zimbabwe and St. Mary’s Research Ethics Committee, London.

#### Masaka

The MRC DSS approved by the Uganda Virus Research Institute (UVRI) Science and Ethics Committee (SEC) and the Uganda National Council of Science and Technology (UNCST). Study participants provided written consent to participate in any part of the study. 

#### Rakai

The Rakai Community Cohort Survey approved by the UVRI SEC andUNCST. Literate participants provided written consent while those unable to read or write had a witness sign on their behalf.

### Identifying pregnancy periods

For all the sites, in the absence of active pregnancy reporting, pregnancy periods can be identified from the date of birth of a child. This information either comes from a mother-child data link or from a women reporting that she gave birth. All studies apart from Karonga and Africa Centre also collect routine data on current pregnancy status, giving limited information on pregnancy periods that do not result in a live birth. Such pregnancies are harder to identify for a number of reasons: firstly women rarely report a pregnancy in the first trimester; secondly many DSS use proxy respondents so it is possible that they will not know the women in their household is pregnant until sometime past the first trimester. Pregnancies ending in early miscarriage are thus rarely captured. Rakai is a partial exception as they have done routine hCG (human chorionic gonadotropin) testing if the last menstrual period was delayed or the woman was unsure of her pregnancy status [2]. Pregnancies ending in stillbirth may be captured either by asking direct questions about stillbirths since the last DSS round, or by noting reported pregnancies that did not result in a live birth in a later round. However only those DSS which have consistently maintained a short time gap between survey rounds (ideally ≤4 months) can be reasonably certain of interviewing women after the first trimester but before the stillbirth occurs. Early miscarriage and abortion are estimated to make up a fairly small proportion of total time pregnant therefore missing a large fraction of these would be unlikely to affect the results. 

### Analysis methods

Women of reproductive age (15- 49 years old) were eligible for inclusion in the analysis. Person-years of observation for each woman were split into time not-pregnant, pregnant and one year postpartum. For a woman to be included in the analysis she must have had at least two HIV tests, the first of which must have been negative to allow observation of any sero-conversion. Follow-up time started from the date of the first negative test and lasted until exit at the date of their last test or at the date of sero- conversion, if earlier. 

Time between HIV surveillance tests varies across the different sites ranging from annual to three year inter-test intervals; further, a person might miss a surveillance round thus extending the period between tests. For all study sites, the interval between HIV tests is longer than a full gestation pregnancy, and we cannot be sure whether the sero-conversion occurred before, during or after the pregnancy period. To allow for this uncertainty, the analysis was repeated 100 times, each time with the estimated sero-conversion date assigned at a random point between the last negative and first positive dates, rates and crude and adjusted hazard rate ratios (HRR) were calculated using piecewise exponential regression, so that age (grouped into conventional five year age groups), pregnancy status and calendar time could be treated as time-varying factors. Rates and the log of the hazard rate ratios from the imputations were combined using Rubin’s rules [26] to give confidence intervals that reflect the uncertainty about the exact date of sero-conversion. The crude hazard rate ratios converged at around 20 imputations with the adjusted rate ratios taking 30 to 40 imputations to converge to stable values. 

Since the introduction of widespread voluntary counselling and testing and the roll-out of antiretroviral treatment (ART) in sub-Saharan Africa, it is possible that the composition of those who do not consent to test/participate in surveillance has changed, potentially biasing results. For example, people who know they are HIV-positive may be less likely to consent to participate in an HIV surveillance round [17,27,28], this would be especially pertinent for women who are HIV tested in antenatal care (ANC) clinics in the context of prevention of mother-to-child transmission (PMTCT) services. Women who are not pregnant may have less exposure to HIV testing, although community-based HIV testing is becoming more widespread. The possibility of bias after PMTCT programmes were introduced (post-PMTCT period) is addressed by stratifying the data by the pre- and post-PMTCT periods. Post-PMTCT is defined from the point when PMTCT became available and accessible to the populations. In some studies, this time preceded introduction of HIV treatment programmes (Masaka, Rakai and uMkhanyakude). 

Surveillance data from the Kisesa, Masaka, Manicaland and Rakai studies all include a period before PMTCT was widely available at ANC. For Karonga and uMkhanyakude HIV surveillance data are only available after introduction of widespread PMTCT services (table 1). 

**Table 1:** Data available from sites by periods in which availability of PMTCT was low medium and high.

**Site**	**Availability of data by level of PMTCT services**	
	**None/Very low**	**Some/Widespread**
**Karonga**	No data available	2007-2011
**Kisesa**	1994-May2007	June 2007-2010
**Manicaland**	1998-2008	No data available
**Masaka**	1989-Mar2002	April 2002-2010
**Rakai**	1999-May2004	June 2004-2011
**uMkhanyakude**	No data available	2001-2011

This paper investigates the risk of HIV acquisition during pregnancy and in the postpartum period in both the pre- and post-PMTCT period. For the pre-PMTCT period person years are censored at date of last test prior to widespread PMTCT. The post-PMTCT period includes all the person years from the date PMTCT began to be more widely available in each site. 

## Results

Table 2 and Table 3 show site specific and pooled rates before and after introduction of PMTCT. Overall there were 2628 sero-conversions and a total of 178 thousand person years contributing to the analysis. The number of person years and sero-conversions in the pre-PMTCT period is lower than in the post-period, partly due to fewer study sites contributing and partly due to the strict censoring at last test prior to PMTCT beginning in each site. uMkhanyakude contributes around two-thirds of the sero-conversions in the post-PMTCT period, but only a sixth of the person-years due to its relatively high incidence and low fertility setting. Karonga only provides a small number of sero-conversions and few person-years due to a shorter follow-up time. Using the mean of the imputation runs 304 sero-conversions occurred in the 25,000 person years spent pregnant. 

**Table 2:** Sero-conversion and person years contributing to the analysis for each period (mean of imputation runs).

		All* (Six Sites)				**Pre PMTCT (Four sites)**				**Post PMTCT (Five sites)**		
	**Pregnancy and Maternity Status**	**SC**	**1000 PY**	**Rate /1000**		**SC**	**1000 PY**	**Rate /1000**		**SC**	**1000 PY**	**Rate /1000**
**Maternity Status**												
	Not pregnant	1861	121.10	15.37		271	28.33	9.57		1245	68.43	18.20
	Pregnant	304	24.75	12.28		62	6.65	10.32		169	12.52	13.46
	<1 year post partum	463	31.49	14.70		81	8.30	8.87		262	16.52	15.86
**Pregnancy Status**												
	Not pregnant	2324	152.57	15.23		345	36.63	9.41		1507	84.95	17.74
	Pregnant	304	24.75	12.28		69	6.65	10.32		169	12.52	13.46

Note that the uMkhanyakude and Karonga site only contributes to the post PMTCT period.

^*^ Note that all is not the sum of pre and post- PMTCT due to the nature of censoring for the pre-PMTCT group.

**Table 3:** Number of Sero-conversions (SC) and person years (PY) contributing to the analysis for each site by period (mean of imputation runs).

		**Karonga**				**Kisesa**				**Manicaland**				**Masaka**				**Rakai**				**uMkhanyakude**		
	**Pregnancy and Maternity Status**	**SC**	**1000 PY**	**Rate per 1000 PY**		**SC**	**1000 PY**	**Rate per 1000 PY**		**SC**	**1000 PY**	**Rate per 1000 PY**		**SC**	**1000 PY**	**Rate per 1000 PY**		**SC**	**1000 PY**	**Rate per 1000 PY**		**SC**	**1000 PY**	**Rate per 1000 PY**
**Pre PMTCT**																								
	Not pregnant	-	-	-		57	6.30	9.06		84	6.08	13.80		65	9.16	7.05		65	6.80	9.57		-	-	-
	Pregnant	-	-	-		12	2.06	5.85		10	0.55	18.31		11	1.88	5.84		36	2.18	16.55		-	-	-
	<1 year post partum	-	-	-		17	2.64	6.50		7	0.74	9.99		13	2.30	5.54		36	2.63	13.88		-	-	-
**Post PMTCT**																								
	Not pregnant	34	8.15	4.22		19	3.13	5.93		41	3.70	11.10		83	13.43	6.16		230	20.43	11.26		841	19.70	42.69
	Pregnant	9	1.73	4.99		3	0.65	3.99		1	0.34	1.75		16	2.42	6.44		36	5.31	6.80		105	2.04	51.43
	<1 year post partum	9	2.46	3.55		6	0.94	6.77		2	0.44	4.24		20	3.22	6.19		80	6.64	11.98		146	2.75	52.88
**All Years**																								
	Not pregnant	34	8.15	4.22		170	17.33	9.78		241	16.95	14.20		175	25.51	6.88		401	33.44	11.99		841	19.70	42.69
	Pregnant	9	1.73	4.99		36	5.19	6.84		23	1.55	14.65		31	4.84	6.40		100	9.40	10.64		105	2.04	51.43
	<1 year post partum	9	2.46	3.55		53	6.52	8.10		39	2.11	18.46		38	6.17	6.20		178	11.47	15.55		146	2.75	52.88

Note that each site covers a different period of calendar time.

In the pooled data, the crude analysis showed no evidence of different risks of HIV acquisition between pregnant or postpartum women and non-pregnant women in the pre-PMTCT era (Table 4). After adjusting for age, the rate ratios showed a protective effect for pregnant and postpartum women compared to those who were not pregnant, although this did not reach statistical significance for pregnant women (HRR 0.85, 95%CI 0.63-1.13 and HHR 0.75 95%CI 0.57-0.98, respectively). In the post-PMTCT period, there was evidence of a protective effect against HIV acquisition in both pregnant and postpartum women when adjusted for age (HHR 0.60, 95%CI 0.50-0.71 and HHR 0.71 95%CI 0.62-0.82 respectively (Table 5)). After adjusting for study site the evidence became of borderline statistical significance for postpartum women. 

**Table 4:** Incident rate ratio comparing pregnancy status for pre PMTCT period.

**Pregnancy and Maternity Status**	**Crude**			**Adjusted Age**			**Adjusted Age and Site**	
	**HRR**	**95% CI**		**HRR**	**95% CI**		**HRR**	**95% CI**
**Maternity Status**								
**Not pregnant**	1			1			1	
**Pregnant**	1.08	(0.82-1.42)		0.85	(0.64-1.13)		0.89	(0.67-1.19)
**<1 year post partum**	0.93	(0.71-1.21)		0.75	(0.57-0.98)		0.78	(0.59-1.03)
**Pregnancy Status**								
**Not pregnant**	1			1			1	
**Pregnant**	1.10	(0.84-1.43)		0.93	(0.71-1.22)		0.96	(0.73-1.26)

**Table 5:** Incident rate ratio comparing pregnancy status for the period post widespread PMTCT.

**Pregnancy and Maternity Status**	**Crude**			**Adjusted Age**			**Adjusted Age and Site**	
	**HRR**	**95% CI**		**HRR**	**95% CI**		**HRR**	**95% CI**
**Maternity Status**								
**Not pregnant**	1			1			1	
**Pregnant**	0.74	(0.63-0.87)		0.60	(0.50-0.71)		0.75	(0.64-0.89)
**<1 year post partum**	0.87	(0.76-1.00)		0.71	(0.62-0.82)		0.89	(0.77-1.02)
**Pregnancy Status**								
**Not pregnant**	1			1			1	
**Pregnant**	0.76	(0.64-0.89)		0.65	(0.55-0.76)		0.77	(0.65-0.91)

Combining all data from all periods gave results very similar to those in the post-PMTCT period: with a rate ratio comparing pregnant to non-pregnant women adjusted by age of 0.69 (95%CI 0.61-0.78), indicating a lower HIV acquisition risk during pregnancy (Table 6). This effect remained when adjusting by study site. 

**Table 6:** Incident rate ratio comparing pregnancy status for all periods.

**Pregnancy and Maternity Status**	**Crude**			**Adjusted Age**			**Adjusted Age and Site**	
	**HRR**	**95% CI**		**HRR**	**95% CI**		**HRR**	**95% CI**
**Maternity Status**								
**Not pregnant**	1			1			1	
**Pregnant**	0.80	(0.71-0.90)		0.64	(0.57-0.73)		0.77	(0.68-0.88)
**<1 year post partum**	0.96	(0.86-1.06)		0.78	(0.70-0.87)		0.92	(0.84-1. 03)
**Pregnancy Status**								
**Not pregnant**	1			1			1	
**Pregnant**	0.81	(0.71-0.91)		0.69	(0.61-0.78)		0.79	(0.70-0.89)

There was evidence of an interaction between age and pregnancy status indicating that the protective effect did not apply to the 15-24 year old age group (all periods pooled HRR 0.84 95%CI 0.50-1.41), this effect remained significant excluding the uMkhayakude which contributes the most data. There was no evidence of interaction between pregnancy status and study site. The analysis was repeated on individual site data combining the pre- and post-PMTCT periods (Table 7); both the Kisesa and Rakai studies individually showed a significant decrease in HIV acquisition rates comparing pregnant to non-pregnant women when adjusted for age (HRR 0.57, 95%CI 0.37-0.87 and HRR 0.71 , 95%CI 0.57-0.89 respectively). Masaka and Manicaland showed a non-significant decrease in HIV acquisition, Karonga and Africa Centre showed no evidence for any difference between HIV acquisitions in pregnant women compared to non-pregnant women; however, the confidence intervals in Karonga are very wide due to the small sample. Kisesa showed a significant decrease and Masaka a borderline significant decrease in HIV acquisition in postpartum compared to non-pregnant women; the other sites showed no evidence for any difference between the two groups.

**Table 7:** Incident rate ratio adjusted for age comparing pregnancy status for all periods by study site.

	**Karonga**		**Kisesa**		**Manicaland**		**Masaka**		**Rakai**		**uMkhanyakude**	
**Pregnancy and Maternity Status**	**HRR**	**95% CI**	**HRR**	**95% CI**	**HRR**	**95% CI**	**HRR**	**95% CI**	**HRR**	**95% CI**	**HRR**	**95% CI**
**Maternity Status**												
**Not pregnant**	1		1		1		1				1	
**Pregnant**	1.17	(0.49-2.79)	0.70	(0.47-1.03)	1.02	(0.62-1.68)	0.93	(0.62-1.39)	0.89	(0.71-1.11)	1.20	(0.97-1.48)
**<1 year post partum**	0.82	(0.33-2.04)	0.83	(0.60-1.14)	1.29	(0.90-1.86)	0.90	(0.63-1.29)	1.30	(1.08-1.55)	1.24	(1.03-1.48)
**Pregnancy Status**												
**Not pregnant**	1		1		1		1		1		1	
**Pregnant**	1.21	(0.51-2.86)	0.66	(0.43-0.99)	0.98	(0.60-1.62)	0.95	(0.64-1.40)	0.82	(0.66-1.03)	1.17	(0.95-1.44)
**Adjusted**												
	**Karonga**		**Kisesa**		**Manicaland**		**Masaka**		**Rakai**		**uMkhanyakude**	
**Pregnancy and Maternity Status**	**HRR**	**95% CI**	**HRR**	**95% CI**	**HRR**	**95% CI**	**HRR**	**95% CI**	**HRR**	**95% CI**	**HRR**	**95% CI**
**Maternity Status**												
**Not pregnant**	1		1		1		1				1	
**Pregnant**	0.97	(0.40-2.40)	0.56	(0.38-0.84)	0.81	(0.49-1.34)	0.76	(0.50-1.15)	0.73	(0.58-0.92)	0.91	(0.73-1.12)
**<1 year post partum**	0.67	(0.26-1.72)	0.68	(0.48-0.95)	1.04	(0.72-1.50)	0.74	(0.51-1.09)	1.08	(0.89-1.30)	0.94	(0.78-1.13)
**Pregnancy Status**												
**Not pregnant**	1		1		1		1		1		1	
**Pregnant**	1.08	(0.45-2.60)	0.57	(0.37-0.87)	0.80	(0.48-1.33)	0.82	(0.55-1.23)	0.71	(0.57-0.89)	0.92	(0.74-1.13)

## Discussion

This study is the first to look at risk of HIV acquisition during pregnancy at a population level, without restricting the analysis to sexually active women or to sero-discordant couples. These data show some evidence that, in the whole population, pregnant women have a lower risk of HIV acquisition during pregnancy than women who are not pregnant and no evidence that postpartum women have a different risk of HIV acquisition in the first year post-pregnancy compared to non-pregnant women. 

A study by Mugo et al. found an unadjusted rate ratio of 2.34 (95% CI 1.33-4.09) comparing pregnant to non-pregnant women [5], however, this study enrolled sero-discordant couples with at least three reported episodes of vaginal intercourse during the three months prior to screening and who intended to remain a couple and thus were a selected population. In the population overall, pregnancy is more likely to be desired in a stable partnership such as marriage. Being in a stable partnership would imply that the couple have had sex on a frequent basis, and, therefore by the time of a pregnancy, will be more likely to be sero-concordant with their partner. The higher the parity of the birth, the more likely the couple are to be HIV concordant (if the births have occurred within the same partnership) as they will have had a longer period of sexual partnership. Assuming that a higher proportion of pregnant women are in stable partnerships than non-pregnant women, it is likely that a higher proportion of pregnant women have sero-negative partners compared to the non-pregnant women. This is because those already concordant-positive will not be at risk of infection and therefore will be excluded from the analysis. The interaction evidence that the slight protective effect is not seen in the youngest age group might go further to support this theory as they have had less time to become concordant with their partner. Also those who have never had a sexual partner, a relatively large fraction of the under-20 age group, will not be at risk of infection and will not be pregnant. 

Studies of sexually active women are less selective than sero-discordant couple studies but could still be different to those based on the whole population. The definition of sexually active women varies across studies, some exclude all women who report no sexual activity in the intervals between survey rounds, which may cause the exclusion of women who report no sexual activity during or immediately after pregnancy [2], some exclude only women who did not have a partner in the last 12 months [7], and some exclude those who were not sexually active at enrolment [4] with the time reference period left unspecified. If all sexually inactive women in both the non-pregnant and pregnant groups were excluded, differences in the age-specific proportion sexually inactive in the two groups could give rise to spurious results. Pregnant women or those who had recently given birth might be less sexually active due to the pregnancy, especially in cultures where prolonged post-partum abstinence is the cultural norm [29]. Non-pregnant women may be excluded because they have never had a sexual partner – in these two cases the excluded women are at lower risk of infection. But in other cases, exclusion of women retrospectively reporting no recent sexual activity may lead to excluding high risk groups: e.g. women whose marriages have recently broken up due to widowhood and separation (these events occur more frequently among women with HIV positive partners [30]); or women who live apart from their partners because of the nature of their employment [31]. The prospective behaviour of women who have recently experienced a period without sexual activity may also place them at high risk in the near future e.g. at the time of first sex or when acquiring a new partner [32]. 

Using sexually active women from sites in Uganda and Zimbabwe Morrison et al [4] overall found no difference between the pregnant and not pregnant women (HRR 0.56 95% CI 0.30-1.05); however, they did find evidence for an interaction with site; the Zimbabwean site showed a lower risk of HIV acquisition in pregnant women (HR 0.26; 95% CI 0.10-0.68). As with this study they also found some evidence of interaction with age, with no difference in HIV acquisition for younger women (HRR 1.14; 95% CI 0.47-2.80) but a lower risk during pregnancy for older women (HRR 0.37 95% CI 0.13-1.09); however, this was not statistically significant. A further prospective study found no increased risk [7]. Only one prospective study of sexually active women in Uganda found a significant increased risk in HIV transmission during pregnancy (HRR 2.03 95% CI% 1.33-3.11) unadjusted and a similar result after adjusting for covariates [2]. The study shows that when stratifying by age the rate ratio only remains statistically significant for those 15-19 years old, showing a similar age effect to this study and to the study by Morrison et al [4]. The Ugandan study sample of sexually active women [2] came from the same population as the Rakai study in this analysis at an earlier time period but gives an increased risk of HIV acquisition in pregnant women rather than the decreased risk we see in this analysis when using the whole population. 

In this analysis overall, we find no evidence of increased HIV incidence in the post-partum period when compared to non-pregnant and non-postpartum periods. There was some evidence of a decreased risk in this period once adjusted for age; however, statistical significance was lost when also adjusting for site indicating heterogeneity between study sites. To be consistent with studies that found a higher incidence immediately postpartum followed by a decrease over time, we would expect to see a significantly higher incidence in women in the post-partum period than in women who were neither pregnant nor post-partum. There are a number of differences in the studies cited above: those noting an increase in risk are not from the general population but from antenatal clinics or hospital delivery wards, therefore restricting the analysis to women who have given birth, whereas in this study the non-pregnant non-postpartum women may never have given birth or last gave birth a long time ago. Also it is possible, as Leroy et al suggest [3], that studies noting a decrease in incidence over time could be affected by a cohort selection effect whereby high risk sub-groups sero-convert early on, leaving the cohort survivors composed mainly of low risk sub-groups. Finally, the incident rate confidence intervals in these studies either overlapped between groups [3] or are not shown [8]; therefore, the results give weak but inconclusive evidence. Our results here are consistent with a study of sexually active women in Uganda [2] which showed no significant difference in those women breastfeeding compared to those not pregnant and non-lactating.

The major strength of this study is that it is population-based rather than selected from clinics or hospitals, therefore we are able to assess the population risk of HIV transmission during pregnancy. Also we have pooled data from six different study sites that contribute 178,000 person years of data which makes this study much larger than previous studies on this topic. 

Four sites were able to contribute to the pre-PMTCT period, where there is less possibility of bias due to those who know they are infected being less likely to agree to testing, however the results from the pre- and post-analysis were consistent although the pre-PMTCT period did not reach statistical significance because of the limited sample size available. If there was a bias in the post-PMTCT period it would have to be very large to overturn the protective effect of pregnancy shown in this study (HRR 0.65; 95%CI 0.61-0.78) and generate an increased risk of around two as seen in the sero-discordant couple studies. Kisesa, one of the study sites that, on its own, showed a lower risk of HIV acquisition during pregnancy, actually had one of the shortest periods where PMTCT was available, therefore it is less likely to be biased due to differences between pregnant and non-pregnant women knowing their HIV status and their subsequent participation in the surveillance study. 

The major limitation of this analysis is the source of HIV test data from surveillance rounds that may be two or three years apart giving long sero-conversion intervals. Thus, we only know that a woman was pregnant at some point during the interval but do not know if the sero-conversion occurred before, during, or immediately after, the pregnancy. The imputation method used enables us to allow for this uncertainty and to generate confidence intervals to reflect this. When restricting the analysis to shorter sero-conversion intervals, the results did not change. The identification of a pregnancy interval may also lead to uncertainty, pregnancies that end in miscarriage are rarely reported in these studies, stillbirths are also often not captured, therefore some of the pregnancy person years will be miscategorised as not pregnant. However these person-years will be small in comparison with all the pregnancies identified by live births and those pregnancies that are captured with a pregnancy report. HIV infected women suffer more miscarriages and stillbirths than their uninfected counterparts [33] however this decrease in viability of pregnancy is associated with longer duration of infection [34], there are no studies that suggest an association of sero-conversion with pregnancy loss.

Although there might be immunological reasons for increased risk of HIV acquisition during pregnancy, at a population level this study indicates a lower risk of HIV acquisition in pregnant women. This is probably due to a range of socio-behavioural characteristics of women and their partners that determine which women are most likely to become pregnant and which women will become infected, and these factors could be investigated in further analyses.

This study furthers understanding of the population risk of HIV acquisition during pregnancy and in the first year postpartum. The results can inform modellers and help health care providers with decisions on the kinds of interventions that would do most to help prevent the spread of HIV. 
